# Supplementary material for: Analysis of meiosis in Pristionchus pacificus reveals plasticity in homolog pairing and synapsis in the nematode lineage
Source: eLife. 2021 Aug 24;10:e70990. doi: 10.7554/eLife.70990 (PMC8455136; doi:10.7554/eLife.70990)
Supplement: Figure 1—figure supplement 1—source data 1. — See the figure supplement legend for details. [file elife-70990-fig1-figsupp1-data1.docx]

>Ppa_SPO-11

MFSITNAHRRMLSNNQRKMIAHEVVLSSVRHSPSLNPSHSLDCNALTDIPLSRISKGKSEKTRPMIQHSSTMDVMEWIVEDLITQIFKGRKLKIRSSLDESCQQLHYNKSTLRKLAVNSRNISQIYQLIVKSQKSTLRDLYYDHKHLFARQDSLNRSVSDLCQVLNMQRCQVNVISCSKGLVFGRLCLTPFKEKEDITPLDCSKEPILISESIICHIPSSSARFILVVEKDATFQKLIDDNFFELYPRSILVTGKGYPDLITRQFLSRLIDHLNIPIHGLFDADPHGMEIFLTYKYGSSSGRVEGRDAIVPSMKWMGLKPSERDSIPIAHNQLLAMSNVDYKKSRRVKRRAATLDEDDIVRELDCFEANPFKMELEALSSAGHAFLPRFYISPRLKHILAD

>Cele_SPO-11

MYEYSFNPNIDHEPGSVESQQSTIYSDSDDSDDSFLDDEVIPPKEQAMRKIEFALADIKRQMDNKEKSLTLRISTSKSHFCLRYTAKRKGKLDRDLHCLHQVYDLLENDKRSTKRELYYEHKAVYGNQKYLDSSIKSICELLNESRANLNILSCGRGIIRGAITFLVENVGVIDARVQEVLITDALLFSNIISEADFILVVEKDTTFQKLMDENFQAMFPRGILATSKGYPDIATRNVLKMLSEKRKFPIYGLFDADPHGIEIYLTYKYGPTKEFAEGRGAFVPTIEWIGLFPTDFHRFTIDQSQCLPLVRTDFVKIEKMIPRSIQLGEIVVTRELDWMIQNKFKMELESINMCGQEYMARFLIAPRVMSIEKEIPIQPETIINEYHEDSQCSLSTDDDREAKDDDYIDSDAEEKFQNMIDNDSD

>Hsap_SPO11

MAFAPMGPEASFFDVLDRHRESLLAALRRGGREPPTGGSRLASSSEVLASIENIIQDIITSLARNEAPAFTIDNRSSWENIKFEDSVGLQMVSHCTTRKIKSDSPKSAQKFSLILKILSMIYKLVQSNTYATKRDIYYTDSQLFGNQTVVDNIINDISCMLKVSRRSLHILSTSKGLIAGNLRYIEEDGTKVNCTCGATAVAVPSNIQGIRNLVTDAKFVLIVEKDATFQRLLDDNFCNKLSPCIMITGKGVPDLNTRLLVKKLWDTFHVPVFTLVDADPHGIEIMCIYKYGSMSMSFEAHHLTVPAIRWLGLLPSDLKRLNVPKDSLIPLTKRDQMKLDSILRRPYVTCQPFWRKEMEIMADSKMKAEIQALTFLSSDYLSRVYLPNKLKFGGWI

>Mmus_Spo11

MAFAPMGPEASFFDALDRHRASLLAMVKRGAGETPAGATRVASSSEVLTAIENIIQDIIKSLARNEVPAFTIDNRSSWENIMFDDSVGLRMIPQCTTRKIRSDSPKSVKKFALILKVLSMIYKLIQSDTYATKRDIYYTDSQLFGNQAAVDSAIDDISCMLKVPRRSLHVLSTSKGLIAGNLRYMEEDGTRVQCTCSATATAVPTNIQGMQHLITDAKFLLIVEKDATFQRLLDDNFCSRMSPCIMVTGKGVPDLNTRLLVKKLWDTFHIPVFTLVDADPYGIEIMCIYKYGSMSMSFEAHNLTIPTIRWLGLLPSDIQRLNIPKDSLIPLTKHDQMKLDSILKRPYITYQPLWKKELEMMADSKMKAEIQALTLLSSDYLSRVYLPNKLRFGGWI

>Athal_Spo11-1

MEGKFAISESTNLLQRIKDFTQSVVVDLAEGRSPKISINQFRNYCMNPEADCLCSSDKPKGQEIFTLKKEPQTYRIDMLLRVLLIVQQLLQENRHASKRDIYYMHPSAFKAQSIVDRAIGDICILFQCSRYNLNVVSVGNGLVMGWLKFREAGRKFDCLNSLNTAYPVPVLVEEVEDIVSLAEYILVVEKETVFQRLANDMFCKTNRCIVITGRGYPDVSTRRFLRLLMEKLHLPVHCLVDCDPYGFEILATYRFGSMQMAYDIESLRAPDMKWLGAFPSDSEVYSVPKQCLLPLTEEDKKRTEAMLLRCYLKREMPQWRLELETMLKRGVKFEIEALSVHSLSFLSEVYIPSKIRREVSSP

>Athal_Spo11-2

MEESSGLSSMKFFSDQHLSYADILLPHEARARIEVSVLNLLRILNSPDPAISDLSLINRKRSNSCINKGILTDVSYIFLSTSFTKSSLTNAKTAKAFVRVWKVMEICFQILLQEKRVTQRELFYKLLCDSPDYFSSQIEVNRSVQDVVALLRCSRYSLGIMASSRGLVAGRLFLQEPGKEAVDCSACGSSGFAITGDLNLLDNTIMRTDARYIIIVEKHAIFHRLVEDRVFNHIPCVFITAKGYPDIATRFFLHRMSTTFPDLPILVLVDWNPAGLAILCTFKFGSIGMGLEAYRYACNVKWIGLRGDDLNLIPEESLVPLKPKDSQIAKSLLSSKILQENYIEELSLMVQTGKRAEIEALYCHGYNYLGKYIATKIVQGKYI

>Scer_Spo11

MALEGLRKKYKTRQELVKALTPKRRSIHLNSNGHSNGTPCSNADVLAHIKHFLSLAANSLEQHQQPISIVFQNKKKKGDTSSPDIHTTLDFPLNGPHLCTHQFKLKRCAILLNLLKVVMEKLPLGKNTTVRDIFYSNVELFQRQANVVQWLDVIRFNFKLSPRKSLNIIPAQKGLVYSPFPIDIYDNILTCENEPKMQKQTIFPGKPCLIPFFQDDAVIKLGTTSMCNIVIVEKEAVFTKLVNNYHKLSTNTMLITGKGFPDFLTRLFLKKLEQYCSKLISDCSIFTDADPYGISIALNYTHSNERNAYICTMANYKGIRITQVLAQNNEVHNKSIQLLSLNQRDYSLAKNLIASLTANSWDIATSPLKNVIIECQREIFFQKKAEMNEIDARIFEYK

>Spom_Rec12

MNSNDKKKVVRSWIEQFVHDFVEQLSKPTKDSVNVALKRRKHNSWNGSLDSKANERQKVKVFSFPRNETTIAQLFRVLDCVHEAVISDTVITKRDIYYRDVDLFKRQTVVDELLGDISNTIGCSRSDLNVEASAKGLVFGSIHIALENGTVITATKPLLISHHRISSITSTAKWVLVIEKEAVFQTLTEEALADTIIVTAKGFPDLMTRKFLVKLAKALPDAKFFGIFDWDPHGLCIYSCFKYGSNAYSHEPHSQLRNLQLLGPLYEDIFNKNQEFSLKLNKRDIKMITTLLQFEGFQKEPVVREQLQRMLFIQKKAEIQAILEFPSWIKGKLADADKSGKHSVR

>Dmel_MEI-W68

MDEFSENIERIALELLSNLVHGNATLSVPRNSSGNVISEYRRVSYNNRGSRHSFCVLIYMLSRVHRLQVRGGSFTVRGLYYDNPLLVRSQSRIAEARLDVCRMLRTSPLSLGILAASKGLVAGDLRLLMTNGDVLDSSLYGGPLTLPTDPEKIDRIETLAEFVLIVEKESVFESLLSRNVFGTFERRFILITGKGYPDCCTRRIVHRLTEENQLAAYILVDADPFGVEIMLVYRHGSKSMSFSSQGLTTPALRWIGLHPSEIPALGTGAVALVAGDNKKINDLLARHDLEPGVRQELRMLQDVQLKAEIESVIDFLTDDYIPNKINRNLFL
