# Supplementary material for: Analysis of meiosis in Pristionchus pacificus reveals plasticity in homolog pairing and synapsis in the nematode lineage
Source: eLife. 2021 Aug 24;10:e70990. doi: 10.7554/eLife.70990 (PMC8455136; doi:10.7554/eLife.70990)
Supplement: Figure 1—figure supplement 2—source data 1. — See the figure supplement legend for details. [file elife-70990-fig1-figsupp2-data1.docx]

>Ppa_RAD-51

MSAQMAHVDADVENEENAGLACQSIMSLEQRGFVKGDLNKLKEAGYHTIEAIAFATRKELIAVKGISEQKAERLQTEAYKLCPMGFTTASEMHARRADMVQIRTGSQALDQLLGGGIETGSITELFGEYRTGKSQICHSLAVICQLPIDMGGAEGKCMWIDTENTFRPERIVAAAQRFGMDPSSVLENVAIARCYNSEHQFHLSVAAAAMMSESRYALLIVDSATGLFRADYNGRGELANRQMALGRFMRQLMKLADQFGIAVVITNQVVSQVDGGAMFQADAKKPIGGHIVAHASTTRLGLRKGKGENRVCKVHQSPCLAEGEATFSITTTGIEDGKDN

>Cele_RAD-51

MGQSWGYEGIAKRSLCTHKWLYNLNLHSINLFLPIESKMSAQASRQKKSDQEQRAADQALLNAAIEDNAMEQDENFTVIDKLESSGISSGDISKLKEAGYYTYESLAFTTRRELRNVKGISDQKAEKIMKEAMKFVQMGFTTGAEVHVKRSQLVQIRTGSASLDRLLGGGIETGSITEVYGEYRTGKTQLCHSLAVLCQLPIDMGGGEGKCMYIDTNATFRPERIIAIAQRYNMDSAHVLENIAVARAYNSEHLMALIIRAGAMMSESRYAVVIVDCATAHFRNEYTGRGDLAERQMKLSAFLKCLAKLADEYGVAVIITNQVVAQVDGGASMFQADAKKPIGGHIIAHMSTTRLYLRKGKGENRVAKMVQSPNLPEAEATYSITNHGIEDARED

>Hsap_RAD51

MAMQMQLEANADTSVEEESFGPQPISRLEQCGINANDVKKLEEAGFHTVEAVAYAPKKELINIKGISEAKADKILAEAAKLVPMGFTTATEFHQRRSEIIQITTGSKELDKLLQGGIETGSITEMFGEFRTGKTQICHTLAVTCQLPIDRGGGEGKAMYIDTEGTFRPERLLAVAERYGLSGSDVLDNVAYARAFNTDHQTQLLYQASAMMVESRYALLIVDSATALYRTDYSGRGELSARQMHLARFLRMLLRLADEFGVAVVITNQVVAQVDGAAMFAADPKKPIGGNIIAHASTTRLYLRKGRGETRICKIYDSPCLPEAEAMFAINADGVGDAKD

>Mmus_Rad51

MAMQMQLEASADTSVEEESFGPQPISRLEQCGINANDVKKLEEAGYHTVEAVAYAPKKELINIKGISEAKADKILTEAAKLVPMGFTTATEFHQRRSEIIQITTGSKELDKLLQGGIETGSITEMFGEFRTGKTQICHTLAVTCQLPIDRGGGEGKAMYIDTEGTFRPERLLAVAERYGLSGSDVLDNVAYARGFNTDHQTQLLYQASAMMVESRYALLIVDSATALYRTDYSGRGELSARQMHLARFLRMLLRLADEFGVAVVITNQVVAQVDGAAMFAADPKKPIGGNIIAHASTTRLYLRKGRGETRICKIYDSPCLPEAEAMFAINADGVGDAKD

>Athal_Rad51

MTTMEQRRNQNAVQQQDDEETQHGPFPVEQLQAAGIASVDVKKLRDAGLCTVEGVAYTPRKDLLQIKGISDAKVDKIVEAASKLVPLGFTSASQLHAQRQEIIQITSGSRELDKVLEGGIETGSITELYGEFRSGKTQLCHTLCVTCQLPMDQGGGEGKAMYIDAEGTFRPQRLLQIADRFGLNGADVLENVAYARAYNTDHQSRLLLEAASMMIETRFALLIVDSATALYRTDFSGRGELSARQMHLAKFLRSLQKLADEFGVAVVITNQVVAQVDGSALFAGPQFKPIGGNIMAHATTTRLALRKGRAEERICKVISSPCLPEAEARFQISTEGVTDCKD

>Scer_Rad51

MSQVQEQHISESQLQYGNGSLMSTVPADLSQSVVDGNGNGSSEDIEATNGSGDGGGLQEQAEAQGEMEDEAYDEAALGSFVPIEKLQVNGITMADVKKLRESGLHTAEAVAYAPRKDLLEIKGISEAKADKLLNEAARLVPMGFVTAADFHMRRSELICLTTGSKNLDTLLGGGVETGSITELFGEFRTGKSQLCHTLAVTCQIPLDIGGGEGKCLYIDTEGTFRPVRLVSIAQRFGLDPDDALNNVAYARAYNADHQLRLLDAAAQMMSESRFSLIVVDSVMALYRTDFSGRGELSARQMHLAKFMRALQRLADQFGVAVVVTNQVVAQVDGGMAFNPDPKKPIGGNIMAHSSTTRLGFKKGKGCQRLCKVVDSPCLPEAECVFAIYEDGVGDPREEDE

>Spom_Rhp51

MADTEVEMQVSAADTNNNENGQAQSNYEYDVNVQDEEDEAAAGPMPLQMLEGNGITASDIKKIHEAGYYTVESIAYTPKRQLLLIKGISEAKADKLLGEASKLVPMGFTTATEYHIRRSELITITTGSKQLDTLLQGGVETGSITELFGEFRTGKSQICHTLAVTCQLPIDMGGGEGKCLYIDTEGTFRPVRLLAVADRYGLNGEEVLDNVAYARAYNADHQLELLQQAANMMSESRFSLLVVDSCTALYRTDFSGRGELSARQMHLARFMRTLQRLADEFGIAVVITNQVVAQVDGISFNPDPKKPIGGNILAHSSTTRLSLRKGRGEQRICKIYDSPCLPESEAIFAINSDGVGDPKEIIAPV

>Dmel_Spn-A

MEKLTNVQAQQEEEEEEGPLSVTKLIGGSITAKDIKLLQQASLHTVESVANATKKQLMAIPGLGGGKVEQIITEANKLVPLGFLSARTFYQMRADVVQLSTGSKELDKLLGGGIETGSITEIFGEFRCGKTQLCHTLAVTCQLPISQKGGEGKCMYIDTENTFRPERLAAIAQRYKLNESEVLDNVAFTRAHNSDQQTKLIQMAAGMLFESRYALLIVDSAMALYRSDYIGRGELAARQNHLGLFLRMLQRLADEFGVAVVITNQVTASLDGAPGMFDAKKPIGGHIMAHSSTTRLYLRKGKGETRICKIYDSPCLPESEAMFAILPDGIGDARES
