# Supplementary material for: Analysis of meiosis in Pristionchus pacificus reveals plasticity in homolog pairing and synapsis in the nematode lineage
Source: eLife. 2021 Aug 24;10:e70990. doi: 10.7554/eLife.70990 (PMC8455136; doi:10.7554/eLife.70990)
Supplement: Figure 1—figure supplement 3—source data 1. — See the figure supplement legend for details. [file elife-70990-fig1-figsupp3-data1.docx]

>Ppa_DMC-1

MQAEQEQVVQGNEIDDEEDEELDIQDVDQLQQHGINVAEIKKLQAAGICTIKGIMMTTRKRMCEIKGLSEAKVDKIKEGAAKLMKTGFITALEVCSRRKQVFKISTGSVEFDKLLGGGIESQAITEVFGEFRTGKTQLSHTLCATCQMPNANGFRGGKIIYIDTENTFRPDRLRPICDRFNMDQEAMLENVLYARAYTSDHQMELLDFVAAKFHEELGVFKLLIVDSIMALFRVDFSGRGELAERQQKLAQMLSRLQKIAEEYNVAVFISNQMTADPGAGMTFQADPKKPIGGHILAHASTTRIMLKKGRAETRIAKIYDSPDMPENEATFAIAAGGIVDAKD

>Hsap_DMC1

MKEDQVVAEEPGFQDEEESLFQDIDLLQKHGINVADIKKLKSVGICTIKGIQMTTRRALCNVKGLSEAKVDKIKEAANKLIEPGFLTAFEYSEKRKMVFHITTGSQEFDKLLGGGIESMAITEAFGEFRTGKTQLSHTLCVTAQLPGAGGYPGGKIIFIDTENTFRPDRLRDIADRFNVDHDAVLDNVLYARAYTSEHQMELLDYVAAKFHEEAGIFKLLIIDSIMALFRVDFSGRGELAERQQKLAQMLSRLQKISEEYNVAVFVTNQMTADPGATMTFQADPKKPIGGHILAHASTTRISLRKGRGELRIAKIYDSPEMPENEATFAITAGGIGDAKE

>Mmus_Dmc1

MKEDQVVQEESGFQDDEESLFQDIDLLQKHGINMADIKKLKSVGICTIKGIQMTTRRALCNVKGLSEAKVEKIKEAANKLIEPGFLTAFQYSERRKMVFHITTGSQEFDKLLGGGIESMAITEAFGEFRTGKTQLSHTLCVTAQLPGTGGYSGGKIIFIDTENTFRPDRLRDIADRFNVDHEAVLDNVLYARAYTSEHQMELLDYVAAKFHEEAGIFKLLIIDSIMALFRVDFSGRGELAERQQKLAQMLSRLQKISEEYNVAVFVTNQMTADPGATMTFQADPKKPIGGHILAHASTTRISLRKGRGELRIAKIYDSPEMPENEATFAITAGGIGDAKE

>Athal_Dmc1

MMASLKAEETSQMQLVEREENDEDEDLFEMIDKLIAQGINAGDVKKLQEAGIHTCNGLMMHTKKNLTGIKGLSEAKVDKICEAAEKIVNFGYMTGSDALIKRKSVVKITTGCQALDDLLGGGIETSAITEAFGEFRSGKTQLAHTLCVTTQLPTNMKGGNGKVAYIDTEGTFRPDRIVPIAERFGMDPGAVLDNIIYARAYTYEHQYNLLLGLAAKMSEEPFRILIVDSIIALFRVDFTGRGELADRQQKLAQMLSRLIKIAEEFNVAVYMTNQVIADPGGGMFISDPKKPAGGHVLAHAATIRLLFRKGKGDTRVCKVYDAPNLAEAEASFQITQGGIADAKD

>Scer_Dmc1

MSVTGTEIDSDTAKNILSVDELQNYGINASDLQKLKSGGIYTVNTVLSTTRRHLCKIKGLSEVKVEKIKEAAGKIIQVGFIPATVQLDIRQRVYSLSTGSKQLDSILGGGIMTMSITEVFGEFRCGKTQMSHTLCVTTQLPREMGGGEGKVAYIDTEGTFRPERIKQIAEGYELDPESCLANVSYARALNSEHQMELVEQLGEELSSGDYRLIVVDSIMANFRVDYCGRGELSERQQKLNQHLFKLNRLAEEFNVAVFLTNQVQSDPGASALFASADGRKPIGGHVLAHASATRILLRKGRGDERVAKLQDSPDMPEKECVYVIGEKGITDSSD`

>Spom_Dmc1

MEEFAEGNDDEQMIFSDIEDLTAHGIGMTDIIKLKQAGVCTVQGVHMSTKRFLLKIKGFSEAKVDKLKEAASKMCPANFSTAMEISQNRKKVWSISTGSEALNGILGGGIQSMSITEVFGEFRCGKTQMSHTLCVTAQLPRDMGGAEGKVAFIDTEGTFRPDRIKAIAERFGVDADQAMENIIVSRAYNSEQQMEYITKLGTIFAEDGQYRLLIVDSIMALFRVDYSGRGELSERQQKLNIMLARLNHISEEFNVAVFVTNQVQADPGAAMMFASNDRKPVGGHVMAHASATRLLLRKGRGEERVAKLNDSPDMPEAECSYVITPGGIADVS
