# Supplementary material for: Analysis of meiosis in Pristionchus pacificus reveals plasticity in homolog pairing and synapsis in the nematode lineage
Source: eLife. 2021 Aug 24;10:e70990. doi: 10.7554/eLife.70990 (PMC8455136; doi:10.7554/eLife.70990)
Supplement: Figure 1—figure supplement 5—source data 1. — See the figure supplement legend for details. [file elife-70990-fig1-figsupp5-data1.docx]

>Ppa_HOP-2

MSKSAAAKAELEAKALTVVPEYMVEQNRPYSAIDVYNNLRQEYGKTLVVKALEHGVSIGVLKEKLLSKQKIFYANQDRLPVADEATLALLDKSIAERSETYASLSAKYKIVHTELASLRSEETTTRLEEMVEESKAEILRLKDRVQLLEVARASTGVDAETEARDMLAKEMGLQKAAVKRKRMACDIIDAIREGSNMPKKQLFDSIGLEIAEGEKV

>Hsap_HOP2

MSKGRAEAAAGAAGILLRYLQEQNRPYSSQDVFGNLQREHGLGKAVVVKTLEQLAQQGKIKEKMYGKQKIYFADQDQFDMVSDADLQVLDGKIVALTAKVQSLQQSCRYMEAELKELSSALTTPEMQKEIQELKKECAGYRERLKNIKAATNHVTPEEKEQVYRERQKYCKEWRKRKRMATELSDAILEGYPKSKKQFFEEVGIETDEDYNVTLPDP

>Mmus_Hop2

MSKSRAEAAAGAPGIILRYLQEQNRPYSAQDVFGNLQKEHGLGKAAVVKALDQLAQEGKIKEKTYGKQKIYFADQNQFDTVSDADLHGLDASIVALTAKVQSLQQSCRHMEAELKELTSALTTPEMQKEIQELKKECAQYTERLKNIKAATNHVTPEEKEKVYRDRQKYCKEWRKRKRMTTELCDAILEGYPKSKKQFFEEVGIETDEDHNVLLPDP

>Athal_Hop2

MAPKSDNTEAIVLNFVNEQNKPLNTQNAADALQKFNLKKTAVQKALDSLADAGKITFKEYGKQKIYIARQDQFEIPNSEELAQMKEDNAKLQEQLQEKKKTISDVESEIKSLQSNLTLEEIQEKDAKLRKEVKEMEEKLVKLREGITLVRPEDKKAVEDMYADKINQWRKRKRMFRDIWDTVTENSPKDVKELKEELGIEYDEDVGLSFQAYADLIQHGKKRPRGQ

>Scerev_Hop2

MAPKKKSNDRAIQAKGSEAEQLIEDYLVSQYKPFSVNDIVQNLHNKVTKTTATKALENLVNEKRIVSKTFGKIIIYSCNEQDTALPSNIDPSQFDFETVLQLRNDLIELERDKSTAKDALDSVTKEPENEDLLTIIENEENELKKIESKLQSLQDDWDPANDEIVKRIMSEDTLLQKEITKRSKICKNLIATIKDSVCPKNMNEFLEEIGFEDI

>Spombe_Meu13

MAKAKEVKAKPIKGEEAEKLVYEYLRKTNRPYSATDVSANLKNVVSKQVAQKALEQLRDTGLIHGKLYGKQSVFVCLQDDLAAATPEELAEMEKQIQELKDEVSVVKTLYKEKCIELQALNNSLSPAEIREKIQSIDKEIEETSSKLESLRNGTVKQISKEAMQKTDKNYDFAKKGFSNRKKMFYDLWHLITDSLENPKQLWEKLGFETEGPIDLN
