# Supplementary material for: Analysis of meiosis in Pristionchus pacificus reveals plasticity in homolog pairing and synapsis in the nematode lineage
Source: eLife. 2021 Aug 24;10:e70990. doi: 10.7554/eLife.70990 (PMC8455136; doi:10.7554/eLife.70990)
Supplement: Figure 1—figure supplement 6—source data 1. — See the figure supplement legend for details. [file elife-70990-fig1-figsupp6-data1.docx]

>Ppa_HOP-1

MPPAVPHGKTTSGSWSATFPVDQESFQDSTLFMKRLLYVAFSQIISSRDLLPSNCFKKRRCENLRLYVFNTVIPEAFECADQLRAVCETIEKGYFRELHLNIFDEKRKADEIIEVYKMGVTYGDDKVSPSVTLSSNEMGRVEIDYKGKEVLKDQTRELLIRLHQITEKLADLPDTAQWTFYILYNDEKTPKGFQARGFNRRPEPYSIAPDAQKLVIGDSSANHHACHFEVTSVFIEDPVEFEELISGDLSAIQNSLRMDSRDTTIDVTTPTKDSNLNDTFDAIPPPSEDADPPKELKRRAKAGGGGVVADLEKAAQKMQIEDKNGNLISASSPEERQPAPKSMSPKKARQTKEKTASPSKTIKASKAVKKTPGNVGVRSSKKRAQTLLI

>Cele_HIM-3

MATKEQIVEHRESEIPIASQWKATFPVDLEIEKNSEMFALRYIKCASAFILDRRGILDEKCFKTRTIDKLLVTAFQSSVPAAKRVSSTFDGLRDAIQQGYLREFAIVFYKKPNEEDINEVFAFRFAYGDEGEIFVSLNNGIDTNESSQELLQAKFVDTDNTKQMFASTIKKLHRCIKKMEPLPQGSDASFRVSYTEKAPKDYTPEGYLLSPMFYTLNQDIRKASIGIVCGGHHKIQMLAASQYLKQDFDLDKTTTLNPNMSIMANQSKRKGRISRDSPYGLSQGITKKNKD

>Hsap_HORMAD1

MATAQLQRTPMSALVFPNKISTEHQSLVLVKRLLAVSVSCITYLRGIFPECAYGTRYLDDLCVKILREDKNCPGSTQLVKWMLGCYDALQKKYLRMVVLAVYTNPEDPQTISECYQFKFKYTNNGPLMDFISKNQSNESSMLSTDTKKASILLIRKIYILMQNLGPLPNDVCLTMKLFYYDEVTPPDYQPPGFKDGDCEGVIFEGEPMYLNVGEVSTPFHIFKVKVTTERERMENIDSTILSPKQIKTPFQKILRDKDVEDEQEHYTSDDLDIETKMEEQEKNPASSELEEPSLVCEEDEIMRSKESPDLSISHSQVEQLVNKTSELDMSESKTRSGKVFQNKMANGNQPVKSSKENRKRSQHESGRIVLHHFDSSSQESVPKRRKFSEPKEHI

>Mmus_Hormad1

MATMQLQRTASLSALVFPNKISTEHQSLMFVKRLLAVSVSCITYLRGIFPERAYGTRYLDDLCVKILKEDKNCPGSSQLVKWMLGCYDALQKKYLRMIILAVYTNPGDPQTISECYQFKFKYTKNGPIMDFISKNQNNKSSTTSADTKKASILLIRKIYVLMQNLGPLPNDVCLTMKLFYYDEVTPPDYQPPGFKDGDCEGVIFDGDPTYLNVGEVPTPFHTFRLKVTTEKERMENIDSTILKPKESKTQFEKILMDKDDVEDENHNNFDIKTKMNEQNENSGASEIKEPNLDCKEEETMQFKKSQSPSISHCQVEQLVSKTSELDVSESKTRSGKIFQSKMVNGNNQQGQTSKENRKRSLRQFRKTVLHVLESSQESVLKKRRVSEPKEHT

>Athal_Asy1

MVMAQKLKEAEITEQDSLLLTRNLLRIAIFNISYIRGLFPEKYFNDKSVPALDMKIKKLMPMDAESRRLIDWMEKGVYDALQRKYLKTLMFSICETVDGPMIEEYSFSFSYSDSDSQDVMMNINRTGNKKNGGIFNSTADITPNQMRSSACKMVRTLVQLMRTLDKMPDERTIVMKLLYYDDVTPPDYEPPFFRGCTEDEAQYVWTKNPLRMEIGNVNSKHLVLTLKVKSVLDPCEDENDDMQDDGKSIGPDSVHDDQPSDSDSEISQTQENQFIVAPVEKQDDDDGEVDEDDNTQDPAENEQQLARVKDWINSRHLDTLELTDILANFPDISIVLSEEIMDQLVTEGVLSKTGKDMYIKKRDKTPESEFTFVKEEADGQISPGKSVAPEDYLYMKALYHSLPMKYVTITKLHNMLDGEANQTAVRKLMDRMTQEGYVEASSNRRLGKRVIHSSLTEKKLNEVRKVLATDDMDVDVTETINKTNGPDAKVTADVSTCGGIHSIGSDFTRTKGRSGGMQQNGSVLSEQTISKAGNTPISNKAQPAASRESFAVHGGAVKEAETVNCSQASQDRRGRKTSMVREPILQYSKRQKSQAN

>Scer_Hop1

MSNKQLVKPKTETKTEITTEQSQKLLQTMLTMSFGCLAFLRGLFPDDIFVDQRFVPEKVEKNYNKQNTSQNNSIKIKTLIRGKSAQADLLLDWLEKGVFKSIRLKCLKALSLGIFLEDPTDLLENYIFSFDYDEENNVNINVNLSGNKKGSKNADPENETISLLDSRRMVQQLMRRFIIITQSLEPLPQKKFLTMRLMFNDNVDEDYQPELFKDATFDKRATLKVPTNLDNDAIDVGTLNTKHHKVALSVLSAATSSMEKAGNTNFIRVDPFDLILQQQEENKLEESVPTKPQNFVTSQTTNVLGNLLNSSQASIQPTQFVSNNPVTGICSCECGLEVPKAATVLKTCKSCRKTLHGICYGNFLHSSIEKCFTCIFGPSLDTKWSKFQDLMMIRKVFRFLVRKKKGFPASITELIDSFINVEDQNNEVKERVAFALFVFFLDETLCLDNGGKPSQTIRYVTSSVLVDVKGIVIPNTRKQLNVNHEYKWHFTTSSPKAESFYQEVLPNSRKQVESWLQDITNLRKVYSEALSPSSTLQELDLNSSLPTQDPIISGQKRRRYDLDEYLEEDKSSVVNDTIKAKDFDESVPAKIRKISVSKKTLKSNW

>Spom_Hop1

MNSYKEEILQTKSDFTLKNLIFFAISTLCYKRALFNENCYKKVNFEIEHFKGADFDCQLKPTVVSLQAGVDKEADSFLEMMKTYIFSLVSMKVPFTVYLIISSQCKSILEDDAVEKEIFSFTINPGSEEKICCESFVSYQRSERFVIKLFLSGNVKTECKDEEKVVQIITKMERFQLSKGEATKAGVFLNTVETKDCMSWLNRGEFKDIVSFYESNNGIAISHCSHAFVPISTEKIMINKESSLFDSQEKIDSQLEKFLQPLKYDEIGSTQILDEQSVEKSLSQGKCEKMQNESRGLREIKNNNPCEEVKKSNWLKKNISGSDKVDKAEKKKALLNCECGDSTEDSEMFQCERCDGWVHCACYGFESDSDPRQPNQLLCYTCLLVDSESSLYDRMTMLVAYRRAIRCIWASEYQGFQKLAARLNCSYADAKRIEERLVNENIIYKEKKRKWIYFTNKSPEMVSYLREKYFTPSRWISHLNFQNYRQENQRVNMRSFLRPERMEVIERPKKVSKTSNTKETDTMKPLRI
