# Supplementary material for: Analysis of meiosis in Pristionchus pacificus reveals plasticity in homolog pairing and synapsis in the nematode lineage
Source: eLife. 2021 Aug 24;10:e70990. doi: 10.7554/eLife.70990 (PMC8455136; doi:10.7554/eLife.70990)
Supplement: Figure 1—figure supplement 7—source data 1. — See the figure supplement legend for details. [file elife-70990-fig1-figsupp7-data1.docx]

>Ppa_SYP-4

MPLWEIDFSDIDSETEVPDDIDGDEDLPEGYNEEFEKVDDFIHDNLVRAKEGNDVDMIKFVVEQGILRYTRLGLKKEVLDKQIWITKQLGKGNVQEMIADQKKKELELKNRVASLRAERDFKKARLEAYEEEKARREDLRQTFIGYSEDLDRQLENAPWLLEKQKKAARRKELEDEIKSLQSDLAKIDLIEERNRIAGKFLAQKLAELYIKREKLFKQLSEEKTSVKKLEEEKRSRRFMEESSQMDCTQAILDGDLDDEETLMKMAKHYKIDTQNLNVPISQMTQESQPTQSICRKRAQSPAESTASSEANDSMSESGEPTAAVFPPPTKKVSIVEPIHNPDESVDERAAQIGANNETNLDESCMEEDDQNGDKSQPEQLETDEREEDDEGREEEEEYREEEEEHAEQHEQEESPTSQESHATGASQESVAYAPDEMVEEETDNNDGFRRPSNPAKVDFEENEDNLEAETSFNPEMILNISANSNDPGGDFFDMMNNQATHKRAVADANGSSPSVGARDNDFMSMFGGGGGGDTGAPVDDATSFSFNFGGADANETNGGGGGGGGEFNFFGF

>Celeg_SYP-4

MSFPTLQVRPNEKNPKVLRCHEFLRQSNQLVERNMINTEGRKMLAKLLTKMVSEAKGIYALDERRAELAKSGKLQEKLKSAYQTSLTVMEDRLHIAELSRQVQEKKLTVQQKYYECDDLRKSVQETNTEARRVQEINEQRLVDTTRATEELIVKSASILKNLENSSDVHKLRAMEEEKRILEEVVEGLRDQKKCLADDIRTKKATLKAESKKSFEKTVIECAKHYKLHQSLMPKLDKSKRSLETLKDEEENRRICGSLSLDETMQFDRSFILASMDKTNKPEKAVQQLTSKSSEVSITPQISETAKSSFSKEPIPTQHPMEVGEEPVIEHSAEESVHIEPPNVTEIREVVPQITIQPTRQDSVVQRDAEELDTSAVPSPQPESILDEPEEEEEELSNHENHADQSMDVMEVDQEVPEESPMEVGEQEEIEQPSVFKDILATEQAALSQEQEPEIVEKQADNDVQFVDDQQVGAQLDVEDEEEVMSENGSNKSNNFSFNFFGNSKGTSAGEGGGEGNFDFNFDGIGAGDDGSNNGGSTGDLDFLNYDGEDEGKGSGNTQSDPFGFASNGNAAGGGGDGSFNFNFDGDGEGGATSGAGGNSTSFFNF

>Cbrig_SYP-4

MSLPSLKKRPNETNSKILRSHEFLRQSTQLIDRSLINAEGSQMLEKMLTKMIAEAQGKYALDEEGAKIAQSGKLLEDLKTTYQTSITVMEERFLLTELARHVQKKLDVNRNYTQCDLLRKATQSSNAEIRRERNVYEKRLQNAKKSALELSTKHACILRLLENSTDVGQLRTIEEDKSILEKEVDLSRSEKIYLAEDIRTKTSTLKAESKKTFQKTIIECAKHYTLFQTLSPKLMSCKTNLERLKNEEESRRACGSLDEMQFDRSFVLASMEKEKSPEKNDLSPPVNLNTSQKVSSRSTSFNESIQQPDQMEVEEFEGDGERNHSKNESAEKINTSRSLLEDITVFPTAPIPRIVDQAPKRRESVVETQRVVPRDPSPEPVDQGEEIEKQTDQSIDVMELDNQEEVEPMEEESQEQTKQGDVKSQKISEKLNSSVTSSFEFPKLNKPAVEVQKSNALKILDKSVDEDDILSENGSNRSTNFSFNFFGNTKSGAGSDVNGVENDTDGNFDFNFGSIGAGDDGLYNGSGGAFDFLGCGGDEEGSTSSTNASDPFGFGASAAGSGGGDTSFNLNFDGDNEGGGASDAGGNSTSFFNF

>Crem_SYP-4

MFPLNKTQIPLHIPVKFQLLFNKSASLHVALGGNMSFPTLLVRPNEKNPKVLRAHEFLRQSNQLVERNLINAEGRKMLAKMLATMVAEVKGIYALDEKRAELTKSGILFEKLKLAHQTSRDVMEDRYSLAELGRQVQEKKLDVQRNYYECDHLRKSVQTTNAEVRRERESNEKRLNKTKTSTETLIAKSASILKLLENSTDVCKLRTMEEEKRILLEEVDELRDEKKALADDIHTKKATLKAESKKPFKKMVIECAKHYALCETLTPKLIKSKKNLQLLKEEEESRRACGSLDETMQFDRSFVLASMDKDTTAENTSRNAAAHNESVQKSSSRSTSFNESIQQNQTMEVEEEQDASHHSENSRDGSTEMPPVPVETTPTPVVVPKPTPAVSKVAPVVAKPAPVVAKPTPVVTKPAPITAKPAPVVTKSYSVATKPAPVAANPPEATPKRRESMVETQRVIPREPSPEPEEEPEEEDANRSVDVMDQGEDQEEDVEPMETEEQPDVVIPPSPAPHENVDIKPSAPEVLDISHQEDSQNDVEMRIDDDEVLSENGSSRSASFNFNFFGNTKGGAGEVVANDTDGNFDFNFGGGDDVSTNGSGGAFDFLNCGGGDEGQSNNNDADPFGFGANTGGTGGGDMSFNFNFDGDNEGGTSLGAGGNSTSFFNF

>Hcon_SYP-4

MSFRTREKGVWRSRDKTTDFLRKRKELEEECRRLSAQVEQNNAVWISRLQTARNQQTRFDELISCSVRIAKQNNEKKAVEALTERLAKLKMECCQFEKERAKLKKELEFQTITPLRVFMVNFAKIALRTYEMQEKLHEARRTEEHLKIEEAERRARVGASEYDVFDITFASQDLALNKTQERAVPTEPPRASLHVLPETAEEETASGGTLNSCESLCSTSPAQPPPASEQDVVLARTNAVKSYVTQQSEAVARETVQSDDNDSGELTRDEDERVSFCSRDFKQAQGMDVEVASNADEECVPLHKDNDRNGTLEERDTMIRDQDVGVGDFSDDDSAIDPEAEDDEMNNSMENGNEKVMEQTEYIEKQDAHSNSFFLPKKSSDKDSNNYSTPISSSQSFVEGQTSAPSSQQSQQNSMNNCAAIPNRKGPSSMMPNSKNPTSMDPSQEADAILNTLLKAPPSPIQEEMAFDFNCSLDNDEFDPTAALNISAHSNDPGADFLSLMESTNEKREKQRQNSSSPAKDFSFSIFGLGGDAASDQGSDKGFAFDFGGTLNGDEDRDGGGFQFDFNASSANDGSGEKTDKGFNLFDF

>Dviv_SYP-4

MELPFVYIDPHEENEDIANVAAFLGQARRIIVNNAVSEEMKRAIFDLASEYIVDVRKYYQlAKEKEALLVKRDADIDEKLHQVERELAEKERINNALRASIEEKKRNIKLSEITIKKSRNKTSDLFRQRKELEEDCKKLDAKVKESNSTLASSLQTAHKQRDRLQMLMEKSPRLAKQITERDAVNALEVRLEQLKSEQSDLKAEEERLRKELELQTATPLRVFIVNFAKITLRTLEMQEKLGEARKVYEQLKAEETERLKSGVCDHEFDASFLSTDYSFKKVNDHGNILRTELMRPAVVSKPVTPVDDGFSTPETLTSGSSSKSSVEPPQTTEEDIIKARTNAVKSYVTQQSETVARNVESSKNGYKDTIYDENERESIYCLENADKSLGVMRTADKETVPSTLENNNIVEIADDDMDVNVSQGVIPEDFSDEDGEEQEVEDDGTIAVENENDDVAINTNYILINWGVNSAERILSQESEDSTAVEGISRMKIDPINTLKIAQMSETSQSILGSDNREKSPISQKFHENSAGRRASMSHRQGCSSQTSENVFNGGSPSPCVDASQEAGAFFSNLLDQPPSPIQDGIPFNFGSGGITEEIDPSVVLNISTHSNDPGGDFLLLMENEKRERDRRSEGSSSAAEFSFSMFGTAADPATSGLGGEFAFDFDAPSQCNERIDSTTFQFNFSEGSNDQEQNNNDGGFNFFDF
