# Supplementary material for: Analysis of meiosis in Pristionchus pacificus reveals plasticity in homolog pairing and synapsis in the nematode lineage
Source: eLife. 2021 Aug 24;10:e70990. doi: 10.7554/eLife.70990 (PMC8455136; doi:10.7554/eLife.70990)
Supplement: Figure 3—source data 1. — Epitope-tagged alleles were generated by in-frame insertion into the endogenous gene loci using CRISPR/Cas9 (see Materials and methods) and homozygosed by selfing of successfully edited progeny. Fidelity of meiotic segregation in the resulting strains was analyzed by counting the frequency of viable embryos and male progeny among whole broods from self-fertilizing hermaphrodites, as indicated. Quantification of wild-type broods is also reported in Figure 5D. [file elife-70990-fig3-data1.docx]

Figure 3—source data 1

| **Genotype** | **% Egg viability (±SD)** | **% Male progeny (±SD)** | **Eggs laid (± SD)** |
| --- | --- | --- | --- |
| **WT** (n=30) | 92.9 (±16.3) | 0.9 (±1.0) | 205 (±55) |
| ***syp-4::HA*** (n=7) | 97.7 (±11.0) | 0.5 (±0.6) | 164 (±15) |
| ***dmc-1::V5*** (n=8) | 95.3 (±4.7) | 1.1 (±1.4) | 170 (±14) |
| ***rad-51::V5*** (n=8) | 107.1 (±18.9) | 1.1 (±1.5) | 157(±15) |
| ***syp-4::HA;***  ***dmc-1::V5; cosa-1::3xFLAG*** (n=8) | 87.0 (±10.6) | 0.4 (±0.6) | 170 (±37) |
