# Supplementary material for: Analysis of meiosis in Pristionchus pacificus reveals plasticity in homolog pairing and synapsis in the nematode lineage
Source: eLife. 2021 Aug 24;10:e70990. doi: 10.7554/eLife.70990 (PMC8455136; doi:10.7554/eLife.70990)
Supplement: Supplementary file 1. [file elife-70990-supp1.docx]

Supplementary file 1

| **Strain** | **El Paco genome**  **reference, v1, 2017** | **Wormbase designation** | **Allele** | **Guide RNA target sequence (PAM sequence underlined)** | **ssDNA template** | **Genotyping primer sequences** |
| --- | --- | --- | --- | --- | --- | --- |
| *cenpc::V5* | *UMM-*  *S71-6.7mRNA-1* | *PPA37734* | *ie1007* | ATGAAGAGATGGATTATAGTA  GG | GGATATTAAAAAAAGGGGGTAAAATTGTACATCG  CAAAAGGATGAGGAGTCATTTAATTCATCTTACTA  TAATCCATCTCTTCAGGTGGAGTCGAGTCCAAGA  AGTGGGTTTGGAATTGGCTTTCCAGATCCGGCT  CCTTCATTCTCTTCATCGAACTCTTCCACTTCCTT  CTCCTTCTGCTCTTTTCTCTTCTTGTT | (f)TTTCTCCAGGAGTGGTTATCG  (r)ACATCGCAAAAGGATGAGGAG |
| *cosa-1::3xflag* | *UMM-*  *S57-3.22mRNA-1* | *PPA23791* | *ie1003* | CTTTATTCTTCATTTTACAGTG  G | GATCATCCCAGGGAGAGAACGACCTACCCAAAC  AGAGATCATAGATCTAATTATCCACTGGGAGCCG  GATCTGATTATAAAGACCATGATGGAGACTATAAG  GATCACGATATTGATTACAAAGACGATGATGATAA  ATAAAATGAAGAATAAAGAGTATTAAATTTATGTTT  GTGTTCGTTTTTGTAATTACTGCTTTG | (f)ACGACCTACCCAAACAGAGA  (r)CGGATGTGGAAAGACGTACC |
| *dmc-1* mutant | *UMM-*  *S442-1.74mRNA-1* | — | *ie1005* | TTCGATAAGCTGCTTGGAGGT  GG | ATTTAATTGTAACATTCAGACATCCAGTTGATCAA  CTAACCTCCGTTATTGCCTGACTTTCGATTCCAC  CactagtgTCCAAGCAGCTTATCGAATTCAACACTT  CCAGTGGAGATTTTAAAGACTTGCTTGCGTCGT  GAACACACT | (f)GGACTCTCGGAGGCTAAAGT  (r)ATTCTCGAGCATTGCTTCCT |
| *dmc-1::V5* | *UMM-*  *S442-1.74mRNA-1* | — | *ie1001* | GCAACGTTTGCCATTGCAGC  AGG | GATAAAACATGATCTTTTGTCCTTCATAATTGATTA  GGTGGAGTCGAGTCCAAGAAGTGGGTTTGGAAT TGGCTTTCCATCCTTTGCATCGACAATTCCTCCT  GC**G**GC**G**ATGGCAAACGTTGCTTCGTTCTCAGGC  ATATC | (f)TGCCTGAGAACGAAGCAACG  (r)ACATGAGATGGCACAAAGGAC |
| *hop-1* | *UMM-*  *S341-6.31mRNA-1* | *PPA10281* |  |  |  |  |
| *rad-51* mutant | *UMM-*  *S442-1.74mRNA-1* | *PPA42255* | *ie1006* | GTCGAGAACGAGGAGAATGC  CGG | ATTTATTGGTTACCTCAAGGGACATGATGGACTG  GCAGGCGAGTCCGGCATACTAGTTTCTCCTCGT  TCTCGACATCAGCGTCGACGTGCGCCATTTGAG CGGACAT | (f)TTCTAGTGACGCGTGTTGTT  (r)ACGAATCCTCGTTGCTGAAG |

| *rad-51::V5* | *UMM-*  *S442-1.74mRNA-1* | *PPA42255* | *ie1008* | GACCTTCTCCATCACAACAA  CGG | GATGGTCAGATAACAGCTATGGAGTGTGTAAATTGGAGAGAGTTAGACGACTAGGTGGAGTCGAGTCCAAGAAGTGGGTTTGGAATTGGCTTTCCATTATCCTTTCCATCTTCGATGCCCGTGGTGGTGATGGAGAAGGTCGCCTCGCCCTCGGCCAGGCATGGAGATTG | (f)GATTCATCTCTGTTTCACAGG  (r)GATCTCGGTTTCTCTCTTGGAAA |
| --- | --- | --- | --- | --- | --- | --- |
| *spo-11* mutant | *UMM-*  *S230-10.9mRNA-1* | *PPA33054* | *ie1004* | ATTCAGAACTTGGCAGAGAT  CGG | ACAAACATCTTTTTGCACGACAGGATTCTCTCAA  TCGATCAGTTTCCGATAACTAGTACTCTGCCAAGTT  CTGAATATGCAAAGATGTCAAGTCAATGTGGTAA GT | (f)GGAAATCCTTCGTTCTCACTATGG  (r)GTCTCAATATCAGACAATTTCATTCCG |
| *syp-4::HA* | *UMM-*  *S245-8.16mRNA-1* | *—* | *ie1002* | GGAGGAGAATTCAACTTCTT  CGG | CAAATGGAGGTGGCGGCGGGGGAGGAGGAGA  GTTTAATTTTTTTGGTTTTTACCCCTACGATGTCC  CAGATTATGCTTAAACCAATTTTTTCGAGCTTGGT  GTAATGTATCCA | (f)CCCGTTGATGATGCTACCAG  (r)GATACATTACACCAAGCTCGAA |
