# Supplementary material for: Analysis of meiosis in Pristionchus pacificus reveals plasticity in homolog pairing and synapsis in the nematode lineage
Source: eLife. 2021 Aug 24;10:e70990. doi: 10.7554/eLife.70990 (PMC8455136; doi:10.7554/eLife.70990)
Supplement: Supplementary file 3. [file elife-70990-supp3.docx]

Supplementary file 3

| **SSLP marker** | **Chromosome** | **Primer sequences** |
| --- | --- | --- |
| L2 | I | (f)GGAATGAGGGTCGTGAGGTA  (r)GCGTTTTCAAAATGAAGCAA |
| L23 | III | (f)AATTCGCAATGACCTATGAGA  (r)ACAAATTCGTCACTCTGGCA |
| L109 | III | (f)GTCCACCGTACCTCTCACCT  (r)TCTTGATCCATCGGAATGCT |
| L100 | IV | (f)CGATCGCCGTTTACGAGG  (r)GTCCCGCATAGCAACCAATA |
| L106 | IV | (f)GAAAGGGACTGCGGAACTC  (r)GTACAGCGTACCGTCCGATT |
| L16 | V | (f)TGAACACTTTCAATGCCTCTTT  (r)GTCGATCTTGATCTTGTCCG |
