# Supplementary material for: Analysis of meiosis in Pristionchus pacificus reveals plasticity in homolog pairing and synapsis in the nematode lineage
Source: eLife. 2021 Aug 24;10:e70990. doi: 10.7554/eLife.70990 (PMC8455136; doi:10.7554/eLife.70990)
Supplement: Supplementary file 4. [file elife-70990-supp4.docx]

Supplementary file 4

| ***Software*** | ***Version*** | ***Relevant parameters*** |
| --- | --- | --- |
| bwa | 2.0pre2-4-gf06fc0c | bwa mem |
| catfasta2phyml | - | -c -f |
| edgeR | 3.18.0 |  |
| freebayes | 1.3.1-17 | --standard_filters |
| HTSeq | 0.6.1 |  |
| IQ-TREE | 1.6.10 | -bb 1000, -bb -m GTR20+G |
| MAFFT | 7.407 | --auto |
| NCBI-BLAST+ | 2.5.0+ | blastp |
| onemap |  | f2 backcross, record(tol=1e-9) |
| OrthoFinder | 2.2.7 | -og |
| PhyloTreePruner | 20150918 | 35 0.9 u |
| Priism | 4.7.1 |  |
| R | 3.5.1 |  |
| softWorx | 7.0.0 |  |
| STAR | 2.5.2 | --outFilterType BySJout --outFilterMultimapNmax 20 --alignSJoverhangMin 8 -alignSJDBoverhangMin 8 --outFilterMismatchNmax 999 --  outFilterMismatchNoverReadLmax 0.04 --alignIntronMin 20 --alignIntronMax 1000000 --alignMatesGapMax 1000000 |
| StringTie | 1.3.3 | -p 10 -m 50 --rf -f 0.1 -a 10 -j 5 -c 2.5 |
| Transdecorder | 3.0.1 |  |
| trimalAl | v1.4.rev15 | -gt 0.8 -st 0.001 -resoverlap 0.75 -seqoverlap 80 |
