# Supplementary material for: Analysis of meiosis in Pristionchus pacificus reveals plasticity in homolog pairing and synapsis in the nematode lineage
Source: eLife. 2021 Aug 24;10:e70990. doi: 10.7554/eLife.70990 (PMC8455136; doi:10.7554/eLife.70990)
Supplement: Supplementary file 5. — No higher-order recombinant chromatids were observed. [file elife-70990-supp5.docx]

Supplementary file 5

|  | **Oocytes (XX)** | | | **Spermatocytes (XO)** | | |
| --- | --- | --- | --- | --- | --- | --- |
|  | crossovers | **count** | map length (cM) | crossovers | **count** | map length (cM) |
| **ChrI** | 2 | 3 | 55.5 | 2 | 1 | 56.6 |
|  | 1 | 45 |  | 1 | 50 |  |
|  | 0 | 44 |  | 0 | 41 |  |
| **ChrII** | 1 | 45 | 49.1 | 1 | 46 | 50.1 |
|  | 0 | 47 |  | 0 | 46 |  |
| **ChrIII** | 1 | 46 | 50.1 | 1 | 50 | 54.6 |
|  | 0 | 46 |  | 0 | 42 |  |
| **ChrIV** | 2 | 0 | 44.74 | 2 | 1 | 45.7 |
|  | 1 | 41 |  | 1 | 40 |  |
|  | 0 | 51 |  | 0 | 51 |  |
| **ChrV** | 2 | 1 | 50.1 | 2 | 0 | 50.1 |
|  | 1 | 44 |  | 1 | 46 |  |
|  | 0 | 47 |  | 0 | 46 |  |
| **ChrX** | 1 | 45 | 49.64 |  |  |  |
|  | 0 | 47 |  |  |  |  |
